# Direct Comparison of Quality of Life in Patients with Allergic Rhinitis Undergoing Sublingual Versus Subcutaneous Immunotherapy

**DOI:** 10.3390/jcm13216397

**Published:** 2024-10-25

**Authors:** Lauren M. Cook, Grace A. Longfellow, Julia C. Kessel, Brian D. Thorp, Adam J. Kimple, Cristine N. Klatt-Cromwell, Brent A. Senior, Charles S. Ebert

**Affiliations:** 1 UNC School of Medicine, University of North Carolina, Chapel Hill, NC 27599, USA; lauren_cook@med.unc.edu (L.M.C.);; 2 Department of Otolaryngology/Head and Neck Surgery, University of North Carolina, Chapel Hill, NC 27599, USAcharles_ebert@med.unc.edu (C.S.E.J.)

**Keywords:** immunotherapy, rhinoconjunctivitis, allergic rhinitis, sublingual immunotherapy, subcutaneous immunotherapy, quality of life, rhinoconjunctivitis quality of life questionnaire (RQLQ)

## Abstract

**Background/Objectives**: Subcutaneous immunotherapy (SCIT) and sublingual immunotherapy (SLIT) are commonly used for allergic rhinitis (AR), yet limited research has directly compared their effects on quality of life (QoL). We aimed to assess QoL differences between SLIT and SCIT recipients. As both forms of immunotherapy have reported benefits, we hypothesize that patients undergoing SLIT and SCIT will have comparable QoL improvements. **Methods**: A cohort study included patients with AR treated with immunotherapy from 2018 to 2022. Patients with obstructive sleep apnea, primary ciliary dyskinesia, cystic fibrosis, vasculitis, rheumatoid arthritis, sarcoidosis, or lupus were excluded. QoL was evaluated using the Rhinoconjunctivitis Quality of Life Questionnaire (RQLQ) at multiple time points. Demographics, additional therapies, and allergen sensitivities were recorded. Data were analyzed using SPSS Statistics. **Results**: A total of 41 participants were eligible for inclusion. Both SLIT and SCIT groups exhibited reductions from baseline RQLQ scores. Within SLIT recipients, 5/7 RQLQ domains significantly improved. SCIT recipients showed significant QoL enhancement in 3/7 domains. The mean difference between SLIT and SCIT cohorts was −0.18 (*p* = 0.57, *d* = −0.18, 95% CI [−0.79, 0.43] at a mean treatment time of 18 months. **Conclusions**: SLIT and SCIT showed comparable RQLQ score reductions after 18 months of therapy, suggesting similar QoL benefits between the two treatment paradigms. Further investigation is needed to explore SLIT vs. SCIT differences in long-term QoL improvements beyond two years.

## 1. Introduction

Patients with allergic rhinitis (AR) often report a diminished quality of life (QoL) due to its negative effects on sleep, social relationships, school or work performance, and family functioning [1]. The primary goal of AR treatment is to mitigate its overall impact on patient’s well-being and QoL. AR is classified based on severity (mild or moderate/severe) and frequency of symptoms (intermittent or persistent) [2]. In most cases, first-line treatment encompasses mono- or combination therapy using antihistamines, whether oral or intranasal (IN), IN saline spray, and the most effective, IN corticosteroids. Despite maximization of these therapies, many patients continue to experience refractory symptoms, prompting alternative treatment options, such as immunotherapy [3].

Immunotherapy (IT) for AR aims to improve immunologic tolerance to allergens by minimizing the production of IgE in response to subsequent exposures. The primary forms of immunotherapy for AR are subcutaneous immunotherapy (SCIT) and sublingual immunotherapy (SLIT). SCIT, which is characterized by a build-up phase over weeks followed by maintenance injections every two to four weeks over a period of years, has historically been the preferred delivery form of IT. Recent advancements have introduced SLIT as a less invasive, and arguably more convenient, alternative. Additionally, the recent literature shows that SLIT is linked to fewer adverse events, including non-fatal and fatal anaphylaxis [4,5,6]. A 2022 meta-analysis found seven direct comparison randomized control trials (RCTs), yet definitive conclusions were hindered by outcome measure variability. Regardless, among these seven trials, none showed significant differences between SCIT and SLIT in commonly measured outcomes, such as symptoms and medication score [5].

There is currently a dearth of information directly comparing SLIT and SCIT head-to-head regarding their relative impacts on QoL. Outcome measures such as the Rhinoconjunctivitis Quality of Life Questionnaire (RQLQ) are used to evaluate QoL in the setting of AR. The RQLQ has a well-established evidence base, having been validated through clinical trials and practical applications for adults aged 17–70 [7,8]. A recent analysis, drawing from 46 RCTs which used the RQLQ for an adjusted indirect comparison, revealed no significant distinction between SCIT and SLIT. Despite these findings, SCIT continues to be more widely utilized [5].

While SLIT offers the convenience of fewer office visits and is a non-invasive approach, as it is available in tablet and aqueous formulations, one of its main challenges is daily patient adherence without direct oversight by a physician. Adherence rates vary within the literature; for example, a 2016 study reported nonadherence rates of 11–50% for SCIT and 3–25% for SLIT [9]. Although cost is a concern due to inconsistencies in insurance coverage for SLIT, some studies provide differing perspectives. A 12-month cost-effectiveness analysis, assuming an 80% compliance rate and efficacies of 70% for SLIT and 80% for SCIT, found the baseline cost per successful treatment outcome to be $1196 for SLIT and $2691 for SCIT. Collectively, this amounts to a yearly cost reduction of $1495 per treated patient, making SLIT the more cost-effective approach per successful outcome from all perspectives [9].

As patients often present with varying degrees of severity and symptomatology, we expect the impact on different domains of the RQLQ to also be concordantly diverse. Presently, the selection of IT largely hinges on the local availability of SCIT vs. SLIT products, as well as physician and patient preference. Analyzing the QoL outcomes of these immunotherapies will potentially guide providers in recommending more effective and tailored treatments for AR patients. We hypothesized that there would be no significant difference in the QoL, as measured by the RQLQ, between patients treated with SCIT and those treated with SLIT.

## 2. Materials and Methods

### 2.1. Study Design and Participants

An IRB-approved, single-institution cohort study was conducted with inclusion of all patients undergoing SCIT or SLIT therapy at the University of North Carolina Hospital System between 2018 and 2022. Eligible participants had a confirmed diagnosis of allergic rhinitis, established through in vitro or skin testing. Individuals were excluded if at least 12 months of treatment was not completed, if RQLQ surveys were not completed correctly (in entirety or if multiple answers (numbers) were marked per question instead of a single number) in at least 3 time points (including baseline), if treatment type was switched (e.g., from SCIT to SLIT) or paused, and if they were diagnosed with obstructive sleep apnea, primary ciliary dyskinesia, cystic fibrosis, vasculitis, rheumatoid arthritis, sarcoidosis, or lupus. Patients completed the Rhinoconjunctivitis Quality of Life Questionnaire (RQLQ) prior to treatment initiation and at four subsequent time points during treatment (6–12 months, 12–18 months, 18–24 months, and 24 months+).

### 2.2. Data Sources and Measurements

Data were extracted from patients’ electronic medical records (EMRs), to capture demographic details and medical histories, including medical comorbidities and concomitant therapy use (e.g., nasal rinse or intranasal steroid) (Table 1). Comprehensive allergy metrics, including average IgE levels and intranasal steroid use, were recorded (Table 1). The RQLQ survey responses, stratified by 7 domains (i.e., activity, sleep, non-nose/eye symptoms, practical problems, nasal symptoms, eye symptoms, and emotional function) were examined pre-treatment and post-treatment at the specified intervals. The RQLQ comprises 28 questions, each scored between 0 and 6, yielding a total score range of 0 to 168 per survey. Lower RQLQ scores denote a higher quality of life, while higher scores indicate a more diminished quality of life. Data were organized and managed using a REDCap database (Research Electronic Data Capture) hosted at the University of North Carolina at Chapel Hill.

### 2.3. Qualitative Variables and Statistical Methods

Data analyses employed Microsoft Excel^®^ (Microsoft Corporation, Redmond, WA, USA) and SPSS Statistics (IBM Corp. Released 2021. IBM SPSS Statistics for Windows, Version 28.0. Armonk, NY, USA: IBM Corp). Descriptive statistics were calculated both individually and comparatively. Both independent and paired *t*-tests were applied, with a two-tailed significance threshold set at an α-value of 0.05 to directly contrast the QoL impact between SLIT and SCIT. To quantify the magnitude of observed differences and changes, Cohen’s *d* and a corresponding confidence interval were employed as a measure of effect. For participants with multiple post-treatment RQLQ scores, the score furthest from treatment initiation was used for analysis.

## 3. Results

### 3.1. Participants and Demographics

A total of 41 subjects met the inclusion and exclusion criteria. The cohort had a mean age of 43 years, with an even gender distribution for SCIT (50% male, 50% female) and near equal distribution for SLIT (42.9% female and 57.1% male) (Table 1). Most participants identified as white (85.4%) and non-Hispanic or Latino (95.1%). Smaller percentages included participants identifying as Black (4.9%) or Asian (4.9%), with 4.9% being of unknown race and ethnicity.

### 3.2. Associated Findings and Allergen Data

An underlying diagnosis of chronic sinusitis was present in almost half of the overall sample (48.8%), while 36.6% had asthma as a comorbidity. A large proportion of study participants used adjunctive therapies, with 75.6% reporting use of IN corticosteroids and 63.4% using nasal irrigation. The SLIT cohort had an 11% higher use of IN corticosteroids than the SCIT group (81% vs. 70%). The two treatment groups had comparable nasal irrigation usage (61.9% for SLIT vs. 65% for SCIT) (Table 2).

Out of the participants, 18 had underwent serum IgE testing, with an average IgE level of 210.0 ng/mL. The SCIT cohort exhibited IgE levels four times higher (336.5 ng/mL) than the SLIT cohort (83.5 ng/mL) (Table 2). In vitro or skin-prick analysis determined the number of positive antigens, correlating to severity category. All individuals were found to have greater than 2 positive antigens via testing, with no subjects classified in the mild category. Most participants fell into the moderate category (90.5% SLIT, 95% SCIT), with fewer in the severe category (9.5% SLIT, 5.0% SCIT). Over half of participants (51.2%) demonstrated an allergy to each tested allergen. Grass pollen was the most common (80.5%), followed by ragweed (65.9%), mold (63.4%), cat dander (58.5), and dust mite (51.2%) (Table 3).

### 3.3. RQLQ Results and Comparison Between SLIT and SCIT

Before immunotherapy initiation, the mean total overall RQLQ score was 2.69. The SLIT group reported a higher mean total score of 2.98 compared to the SCIT group’s score of 2.38. By 6–12 months, total RQLQ scores were reduced by at least 10% from baseline for both the overall cohort and within the SLIT and SCIT treatment groups. At the 12–18-month time point, there was an over 20% reduction in RQLQ scores for all cohorts (Table 4). For participants with multiple post-treatment RQLQ scores (Table 4 and Table 5), the score furthest from treatment initiation was used for analysis.

**Figure 1:**
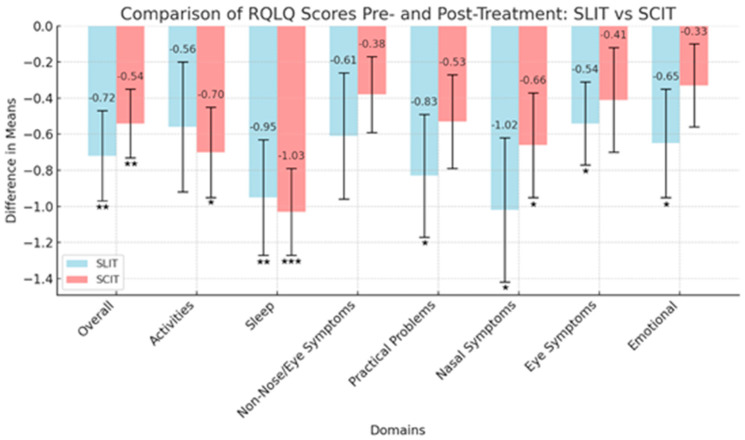
Demonstrates the mean differences in Rhinoconjunctivitis Quality of Life Questionnaire (RQLQ) scores before and after treatment with sublingual immunotherapy (SLIT) and subcutaneous immunotherapy (SCIT). Mean differences were calculated by subtracting pre-treatment scores from post-treatment scores, with negative values indicating improvement. This Figure shows a direct comparison of SLIT vs. SCIT for the seven domains of the RQLQ and overall section means. Error bars represent confidence intervals (listed in Table 6). Notably, SCIT generally shows greater negative mean differences compared to SLIT across most domains, suggesting a greater impact on quality-of-life improvement for this cohort of patients with allergic rhinitis. * denote *p*-values, *** <0.001, ** <0.01, * <0.05.

Overall and domain-specific RQLQ scores pre- and post-treatment were compared and are displayed in Figure 1. Mean differences in RQLQ scores are noted for both the SLIT and SCIT cohorts in the figure below, with more negative values indicating an improved RQLQ score.

### 3.4. Direct Comparison of SLIT Versus SCIT

Paired *t*-test analyses revealed both SCIT and SLIT produced a statistically significant reduction in the total RQLQ score. SLIT resulted in a −0.72 (*p* = 0.009, *d* = −0.63, 95% CI [−1.09, −0.15] reduction in the RQLQ score. Conversely, SCIT resulted in a −0.54 (*p* = 0.009, *d* = −0.65, 95% CI [−1.12 to −0.16] reduction in the RQLQ score. Effect sizes of −0.63 for SLIT and −0.65 for SCIT suggest both IT cohorts had meaningful reductions in RQLQ scores post-treatment. Within the SLIT cohort, statistically significant reductions in RQLQ scores were observed in five out of the seven domains: sleep −0.95 (*p* = 0.007, *d* = −0.66, 95% CI [−1.13, −0.18], practical problems −0.83 (*p* = 0.026, *d* = −0.53, 95% CI [−0.98, −0.06], nasal symptoms −0.54 (*p* = 0.018, *d* = −0.56, 95% CI [−1.02, −0.10], eye symptoms −0.54 (*p* = 0.028, *d* = −0.52, 95% CI [−0.97, −0.06], and emotional function −0.65 (*p* = 0.043, *d* = −0.47, 95% CI [−0.92, −0.01]. For the SCIT cohort, statistically significant reductions in RQLQ score were noted in three domains including: activities −0.70 (*p* = 0.013, *d* = −0.62, 95% CI [−1.09, −0.13], sleep −1.03 (*p* = < 0.001, *d* = −0.96, 95% CI [−1.48, −0.42], and nasal symptoms −0.66 (*p* = 0.032, *d* = −0.52, 95% CI [−0.98, −0.05]. Within this cohort, the non-nose/eye symptoms, practical problems, eye symptoms, and emotional function domains did not show significant changes. Conversely, the SLIT cohort group showed significant changes in all domains except for activities and non-nose/eye symptoms (Table 5).

A direct comparison between SLIT and SCIT utilizing an independent *t*-test revealed that SLIT had a greater overall reduction in average RQLQ post-treatment by 0.18; however, this difference was not statistically significant (*p* = 0.57, *d* = −0.18, 95% CI [−0.79, 0.43]. When SLIT vs. SCIT comparison was extended to all seven individual RQLQ domains, none of the mean differences were statistically significant (Table 6).

## 4. Discussion

Despite maximizing non-immunotherapy options for AR, many patients continue to experience symptom burden, which has been noted in the literature and clinical practice to negatively impact quality of life. Immunotherapy, as a therapeutic option, established itself as the first treatment offering a disease-modifying effect for patients experiencing refractory symptoms.

This observational study aimed to compare quality-of-life improvements between patients undergoing SCIT and SLIT using the Rhinoconjunctivitis Quality of Life Questionnaire (RQLQ). Significant reductions in RQLQ scores after treatment were noted as early as 6–12 months for SCIT and 12–18 months for SLIT. Within these intervals, reductions in RQLQ scores of 46.31% for SLIT and 31.51% for SCIT were observed. Individual analyses of SCIT versus SLIT showed that both treatment modalities were effective in improving the QoL in participants overall. Statistically significant improvements in sleep, non-nose/eye symptoms, practical problems, and nasal symptoms were observed in both the SLIT and SCIT groups. The SLIT treatment group had additional improvements in eye symptoms and emotional function (6 out of 7 domains), while SCIT exhibited additional improvement in activities (5 out of 7 domains). Notably, our study did not identify a statistically significant difference in quality-of-life improvements between SCIT and SLIT overall or for any specific domains.

Both SCIT and SLIT are known to provide a timely onset of benefit, with most patients exhibiting clinical improvement within the first year [10,11,12,13]. Our study yielded comparable results, with the 6–12 month timeframe for SCIT and the 12–18 month period for SLIT indicating the lowest reported RQLQ scores—both encompassing the one-year mark (Table 4). Patients receiving SCIT generally continue to improve while receiving injections and demonstrate stability in their clinical improvement after the cessation of injections. Our cohort plateaued in RQLQ scores after reaching the maximal reduction, although this finding was likely impacted by a decrease in total surveys at later time points.

Our findings are consistent with previous RCT’s, suggesting that both SCIT and SLIT lead to quality-of-life improvements in individuals with AR [14,15,16,17]. A previous prospective analysis of RQLQ score in subjects (*n* = 40) undergoing SCIT and SLIT therapy by Schwanke et al. in 2017 appears to be the most similar study to this, regarding design. Our study builds on their findings but offers additional conclusions likely due to the retention of a more balanced cohort of *n* = 41, SCIT *n* = 21, SLIT *n* = 20, compared to *n* = 40, with 29 participants receiving SCIT and 11 undergoing SLIT (Table 5). Schwanke et al. observed statistically significant improvements in overall RQLQ scores and most domains (except for eye symptoms) at one year within the SCIT group. However, there was no statistically significant improvement in overall RQLQ or any of the domains except for practical functioning at one year within the SLIT group [18]. Additionally, because we were able to collect data beyond 24 months, we were able to provide additional data on the sustained benefits of QoL improvements seen in participants undergoing SLIT and SCIT as seen in Figure 1.

Although SLIT demonstrated a greater reduction in mean difference for overall RQLQ scores compared to SCIT (−0.72 versus −0.54), this was found to be non-significant, as well as the individual reductions for each of the domains (Table 6). SCIT has generally been regarded as slightly more efficacious than SLIT, although existing data varies considerably [19]. Our findings also align with Tie et al.’s meta-analysis results for seven RCT’s, where none showed significant differences between SCIT and SLIT in symptom scores [5].

The concept of minimal important difference (MID) warrants consideration in the context of these study’s findings. Although the data demonstrated substantial percent reductions in overall RQLQ scores, especially the SLIT cohort, those with a significantly lower quality of life may feel a considerable improvement, with relatively small reductions in RQLQ scores. There have been recent efforts, including a 2022 study by Blaiss et al., using an anchor-based method, to derive an MID for mean RQLQ scores in different subsets of patient groups [20]. We endorse these initiatives to more precisely quantify RQLQ scores and better study which subsets of AR patients would benefit from diverse treatment modalities.

Biases in this study include factors altering disease severity at time of survey. Seasonality during survey completion likely had an influence on reported RQLQ scores, with one RCT finding similar scores outside of pollen season between placebo and immunotherapy groups [15]. Also, participants could continue using adjunct therapies such as IN corticosteroids. Thus, RQLQ scores were partially reflective of a combined effect of multiple therapies, although usage was similar between SCIT and SLIT cohorts. Given the specific benefits of SLIT and SCIT observed in individuals with allergic asthma, participants (36.6%) with this condition might have experienced a more pronounced improvement in RQLQ when compared to those without asthma [21]. It would be beneficial for future studies to stratify results based on the presence of asthma and CRS to more effectively parse out the nuances between SLIT and SCIT’s impact on QoL during and after treatment.

This study’s relatively small sample size of 41 patients may have hindered the detection of subtle differences between cohorts. As expected, the standard errors of the means were relatively high when compared to the mean differences, implying some degree of data variability. Additionally, retrospective studies incur limitations in data completeness within medical records. To address this, our study only included patients who completed RQLQ questionnaires for at least 12 months and excluded patients who discontinued immunotherapy treatment. This study mitigated recall bias by ensuring standardized administration of questionnaires before the start of treatment and at subsequent visits.

Another limitation encountered in this study was non-adherence to treatment and loss to follow-up. As mentioned, a consideration when initiating SLIT is patient nonadherence and reduced clinical oversight. In this study, the SLIT cohort started with 21 subjects, yet after 18 months, only seven had documented surveys versus 14 in the SCIT cohort. Additional studies should prioritize higher participant recruitment to ensure the study retains statistical significance at later timepoints, accounting for anticipated dropout rates. It is recommended that patients remain on IT for a minimum of 3 years for maximum clinical benefit [22,23]. Since our study did not follow and capture participants’ RQLQ results during the 3-to-5-year time points, it is possible that scores would have continued to decrease at future time points.

Considering the evolving landscape of treatment options for AR, these findings present patients and clinicians with more data regarding disease-specific quality of life improvement after treatment. These insights should also be contextualized in the setting of varying insurance plan coverage. Blue Cross Blue Shield, a major carrier for patients treated at our institution, does cover FDA-approved SCIT and SLIT formulations (including Oralair^®^, Grastek^®^, Ragwitek^®^, and Odactra^®^), although not comprehensively. Additional research is needed to evaluate patient adherence to treatment regimens and provide more comprehensive guidance for clinical decisions regarding allergic rhinitis management. While our study adds to the body of knowledge concerning the potential therapeutic advantages of SLIT and SCIT, it remains essential to recognize the intrinsic limitations associated with observational data. To definitively assess the comparative efficacy of these treatments, the logical progression would entail a randomized controlled trial.

## Figures and Tables

**Table 1:** Demographics data, *n* (%).

Characteristic	Overall *n* = 41 (100)	SLIT *n* = 21 (51.2)	SCIT *n* = 20 (48.8)
Mean Age at Baseline			
	43	48	39
Sex			
Male	19 (46.3)	9 (42.9)	10 (50.0)
Female	22 (53.7)	12 (57.1)	10 (50.0)
Race			
White	35 (85.4)	20 (95.2)	15 (75.0)
Black	2 (4.9)	0 (0.0)	2 (10.0)
Asian	2 (4.9)	0 (0.0)	2 (10.0)
Other	2 (4.8)	1 (4.8)	1 (5.0)
Ethnicity			
Hispanic/Latino	0 (0.0)	0 (0.0)	0 (0.0)
Not Hispanic/Latino	39 (95.1)	19 (90.5)	20 (100)
Unknown/Declined to Answer	2 (4.9)	2 (9.5)	0 (0.0)

**Table 2:** Associated findings and additional therapies, *n* (%).

Characteristic	Overall *n* = 41 (100)	SLIT *n* = 21 (51.2)	SCIT *n* = 20 (48.8)
Associated Findings			
Chronic sinusitis	20 (48.8)	14 (66.7)	6 (30.0)
Asthma	15 (36.6)	9 (42.9)	6 (30.0)
Total IgE Assessed, *n* (%)	18 (43.9)	10 (47.6)	8 (40.0)
Avg IgE (ng/mL)	210.0	83.5	336.5
Additional Therapies			
Topical Steroid Use	31 (75.6)	17 (81.0)	14 (70.0)
Nasal Irrigation Use	26 (63.4)	13 (61.9)	13 (65.0)

**Table 3:** Allergen data, *n* (%).

Characteristic	Overall *n* = 41 (100)	SLIT *n* = 21 (51.2)	SCIT *n* = 20 (48.8)
Number of Positive Antigens			
Mild (1)	0 (0.0)	0 (0.0)	0 (0.0)
Moderate (2–3)	38 (92.7)	19 (90.5)	19 (95.0)
Severe (4+)	3 (7.3)	2 (9.5)	1 (5.0)
Antigen Type *			
Total Dust Allergy Assessed, *n* (%)	41 (100.0)	21 (100.0)	20 (100.0)
*n* (%) with allergy to dust antigen	21 (51.2)	12 (57.1)	9 (45.0)
Total Mold Allergy Assessed, *n* (%)	39 (95.1)	21 (100.0)	18 (90.0)
*n* (%) with allergy to mold antigen	26 (63.4)	12 (57.1)	14 (77.8)
Total Cat Dander Allergy Assessed, *n* (%)	41 (100.0)	21 (100.0)	20 (100.0)
*n* (%) with allergy to cat dander antigen	24 (58.5)	12 (57.1)	12 (60.0)
Total Grass Pollen Allergy Assessed, *n* (%)	41 (100.0)	21 (100.0)	20 (100.0)
*n* (%) with allergy to grass pollen antigen	33 (80.5)	17 (81.0)	16 (80.0)
Total Ragweed Allergy Assessed, *n* (%)	40 (97.6)	20 (95.2)	20 (100.0)
*n* (%) with allergy to ragweed antigen	27 (65.9)	12 (60.0)	15 (75.0)

* Determined by either in vitro or skin testing.

**Table 4:** Rhinoconjunctivitis quality of life questionnaire results, *n* (%).

Timepoint	Overall *n* = 41 (100)	SLIT *n* = 21 (51.2)	SCIT *n* = 20 (48.8)
Pre-Treatment	*n* = 41 (100)	*n* = 21 (51.2)	*n* = 20 (48.8)
Average per question, Entire Survey (max 6)	2.69	2.98	2.38
Activities	2.87	3.11	2.62
Sleep	2.94	3.13	2.75
Non-nose/eye Sx	2.50	2.71	2.28
Practical Problems	2.88	3.25	2.48
Nasal Sx	3.40	3.81	2.98
Eye Sx	2.29	2.65	1.91
Emotional Sx	2.22	2.55	1.89
Post-treatment *	*n* = 41 (100)	*n* = 21 (51.2)	*n* = 20 (48.8)
Total Assessed from 6 to 12 months after tx, *n* (%)	24 (58.5)	8 (38.1)	16 (80.0)
Average per question, Entire Survey (max 6)	1.98	2.68	1.63
Activities	2.22	3.21	1.73
Sleep	1.93	2.54	1.63
Non-nose/eye Sx	1.81	2.18	1.63
Practical Problems	2.07	2.88	1.66
Nasal Sx	2.55	3.38	2.14
Eye Sx	1.78	2.63	1.36
Emotional Sx	1.68	2.47	1.28
Total Assessed from 12 to 18 months after tx, *n* (%)	20 (48.8)	11 (52.4)	9 (45.0)
Average per question, Entire Survey (max 6)	1.70	1.60	1.83
Activities	1.73	1.64	1.85
Sleep	1.65	1.48	1.85
Non-nose/eye Sx	1.66	1.48	1.89
Practical Problems	1.95	1.94	1.96
Nasal Sx	2.06	1.98	2.17
Eye Sx	1.58	1.59	1.56
Emotional Sx	1.38	1.25	1.53
Total Assessed from 18 to 24 months after tx, *n* (%)	13 (32.5)	4 (19.0)	9 (45.0)
Average per question, Entire Survey (max 6)	1.98	1.95	2.00
Activities	2.08	2.00	2.11
Sleep	1.67	2.08	1.48
Non-nose/eye Sx	1.87	1.68	1.95
Practical Problems	2.05	2.17	2.00
Nasal Sx	2.44	2.19	2.56
Eye Sx	2.10	2.19	2.06
Emotional Sx	1.73	1.63	1.78
Total Assessed from 24+ months after tx, *n* (%)	8 (19.5)	3 (14.3)	5 (25.0)
Average per question, Entire Survey (max 6)	2.29	2.42	2.21
Activities	2.46	2.56	2.40
Sleep	2.29	3.0	1.87
Non-nose/eye Sx	2.34	2.81	2.06
Practical Problems	2.29	1.44	2.80
Nasal Sx	2.88	3.00	2.80
Eye Sx	1.84	1.67	1.95
Emotional Sx	1.94	2.08	1.85

* For participants with numerous post-treatment RQLQ scores, the RQLQ score furthest from treatment initiation was used.

**Table 5:** RQLQ scores pre- and post-treatment, *n* (%).

Group	Difference in Means	Std. Error of the Mean	*p* = x	95% CI	Effect Size
Total cohort *n =* 41 (100)					
Overall, mean change in RQLQ score	−0.63	0.20	0.004	−0.80, −0.016	−0.48
Activities, mean change in score	−0.63	0.271	0.026	−0.67, −0.042	−0.36
Mean, Sleep	−0.99	0.20	<0.001	−1.13, −0.43	−0.78
Mean, Non-nose/eye Sx	−0.50	0.20	0.019	−0.70, −0.06	−0.38
Mean, Practical Problems	−0.68	0.22	0.003	−0.82, −0.17 −0.087, −0.21	−0.49
Mean, Nasal Sx	−0.85	0.25	0.001	−0.087, −0.21	−0.54
Mean, Eye Sx	−0.48	0.18	0.012	−0.73, −0.09	−0.41
Mean, Emotional Sx	−0.49	0.190	0.013	−0.72, −0.09	−0.41
SLIT cohort *n* = 21 (51.2)					
Overall, mean change in RQLQ score	−0.72	0.250	0.009	−1.09, −0.15	−0.63
Activities, mean change in score	−0.56	0.360	0.138	−0.77, 0.11	−0.34
Sleep	−0.95	0.32	0.007	−1.13, −0.18	−0.66
Non-Nose/Eye Symptoms	−0.61	0.35	0.093	−0.83, 0.063	−0.39
Practical Problems	−0.83	0.34	0.026	−0.98, −0.06	−0.53
Nasal Symptoms	−1.02	0.40	0.018	−1.02, −0.10	−0.56
Eye Symptoms	−0.54	0.23	0.028	−0.97, −0.06	−0.52
Emotional	−0.65	0.30	0.043	−0.92, −0.01	−0.47
SCIT cohort *n* = 20 (48.8)					
Overall, mean change in RQLQ score	−0.54	0.19	0.009	−1.12, −0.16	−0.65
Activities, mean change in score	−0.70	0.25	0.013	−1.09, −0.13	−0.62
Sleep	−1.03	0.24	<0.001	−1.48, −0.42	−0.96
Non-Nose/Eye Symptoms	−0.38	0.21	0.094	−0.85, 0.07	−0.39
Practical Problems	−0.53	0.26	0.057	−0.91, 0.01	−0.45
Nasal Symptoms	−0.66	0.29	0.032	−0.98, −0.05	−0.52
Eye Symptoms	−0.41	0.29	0.174	−0.76, 0.14	−0.32
Emotional	−0.33	0.23	0.168	−0.77, 0.13	−0.32

**Table 6:** Comparison of SLIT vs. SCIT RQLQ scores pre- and post-treatment, *n* (%).

	Difference in Means	Std. Error of the Difference	*p* = x	95% CI	Effect Size
Overall, mean change in RQLQ score	−0.18	0.31	0.57	−0.79, 0.434	−0.18
Activities, mean change in score	0.14	0.44	0.75	−0.51, 0.71	0.102
Sleep	0.08	0.40	0.84	−0.55, 0.68	0.06
Non-Nose/Eye Symptoms	−0.23	0.41	0.57	−0.79, 0.44	−0.18
Practical Problems	−0.29	0.43	0.51	−0.82, 0.41	−0.21
Nasal Symptoms	−0.36	0.49	0.47	−0.84, 0.39	−0.23
Eye Symptoms	−0.12	0.37	0.74	−0.72, 0.51	−0.11
Emotional	−0.33	0.38	0.39	−0.88, 0.35	−0.27

## Data Availability

The data presented in this study are available on request from the corresponding author due to patient privacy concerns. Surveys are identified and stored on a password protected document but may be de-identified by the first author and sent to those interested, upon request.
